# Inhibition of Neddylation Suppresses Osteoclast Differentiation and Function In Vitro and Alleviates Osteoporosis In Vivo

**DOI:** 10.3390/biomedicines10102355

**Published:** 2022-09-21

**Authors:** Meng-Huang Wu, Wei-Bin Hsu, Mei-Hsin Chen, Chung-Sheng Shi

**Affiliations:** 1 Department of Orthopaedics, School of Medicine, College of Medicine, Taipei Medical University, Taipei 11031, Taiwan; 2 Department of Orthopedics, Taipei Medical University Hospital, Taipei 11031, Taiwan; 3 TMU Biodesign Center, Taipei Medical University, Taipei 11031, Taiwan; 4 Sports Medicine Center, Chang Gung Memorial Hospital, Puzi 61301, Taiwan; 5 Graduate Institute of Clinical Medical Sciences, College of Medicine, Chang Gung University, Taoyuan 33332, Taiwan; 6 Colon and Rectal Surgery, Department of Surgery, Chiayi Chang Gung Memorial Hospital, Puzi 61301, Taiwan

**Keywords:** neddylation, osteoclast differentiation, osteoporosis, NEDD8-activating enzyme, MLN4924

## Abstract

Neddylation, or the covalent addition of NEDD8 to specific lysine residue of proteins, is a reversible posttranslational modification, which regulates numerous biological functions; however, its involvement and therapeutic significance in osteoporosis remains unknown. Our results revealed that during the soluble receptor activator of nuclear factor-κB ligand (sRANKL)-stimulated osteoclast differentiation, the neddylation and expression of UBA3, the NEDD8-activating enzyme (NAE) catalytic subunit, were dose- and time-dependently upregulated in RAW 264.7 macrophages. UBA3 knockdown for diminishing NAE activity or administering low doses of the NAE inhibitor MLN4924 significantly suppressed sRANKL-stimulated osteoclast differentiation and bone-resorbing activity in the macrophages by inhibiting sRANKL-stimulated neddylation and tumor necrosis factor receptor-associated factor 6 (TRAF6)-activated transforming growth factor-β-activated kinase 1 (TAK1) downstream signaling for diminishing nuclear factor-activated T cells c1 (NFATc1) expression. sRANKL enhanced the interaction of TRAF6 with the neddylated proteins and the polyubiquitination of TRAF6’s lysine 63, which activated TAK1 downstream signaling; however, this process was inhibited by MLN4924. MLN4924 significantly reduced osteoporosis in an ovariectomy- and sRANKL-induced osteoporosis mouse model in vivo. Our novel finding was that NAE-mediated neddylation participates in RANKL-activated TRAF6–TAK1–NFATc1 signaling during osteoclast differentiation and osteoporosis, suggesting that neddylation may be a new target for treating osteoporosis.

## 1. Introduction

Bone remodeling is dynamically mediated by both bone-resorbing osteoclasts and bone-forming osteoblasts; this avoids bone microdamage accumulation and maintains bone strength and mineral homeostasis [1]. An imbalance between bone formation and resorption results in various osteopathic diseases, including osteoporosis [2]. Osteoporosis is characterized by net bone loss, bone tissue deterioration, and bone microarchitecture disruption, causing decreased bone strength and increased bone fragility, thereby leading to fragility fractures [2,3]. The incidence of osteoporosis is high in older people and postmenopausal women [4]; approximately 2–8% of men and 9–38% of women have osteoporosis in developed countries [5]. Osteoclasts are special multinucleated giant cells with tartrate-resistant acid phosphatase (TRAP) expression that form from the self-fusion of hematopoietic monocyte/macrophage lineage cells; they adhere to bone and secrete acid and lytic enzymes for resorbing and remodeling the bone matrix and cause osteoporosis [6]. The survival, generation, and activity of osteoclasts in the bone microenvironment are tightly controlled by cytokines and growth factors produced by osteoblasts, stromal cells, and other cells of hematopoietic origin [7,8]. Among these factors, macrophage colony-stimulating factor (M-CSF) regulates the survival and proliferation of precursor monocytes, and the receptor activator of nuclear factor-κB (RANK) ligand (RANKL) further causes the differentiation of preostoclastic monocytes into active osteoclasts [9,10]. The binding of RANKL with RANK-expressing osteoclasts is the key intracellular signaling for stimulating active osteoclast differentiation through the recruitment of the vital adaptor protein tumor necrosis factor (TNF) receptor-associated factor 6 (TRAF6) to the cytoplasmic tail of RANK [9,11]. TRAF6 exhibits intrinsic ubiquitin E3 activity that autocatalyzes its polyubiquitylation on site-specific lysine 63 (K63) for self-activation [12]; this, in turn, activates and phosphorylates transforming growth factor-β-activated kinase 1 (TAK1). TAK1 regulates the downstream signaling of nuclear factor-κB (NF-κB) and mitogen-activated protein kinases (MAPKs), including c-Jun N-terminal kinase (JNK), p38, and extracellular signal-regulated kinase (ERK), thereby promoting the expression of transcriptional nuclear factor-activated T cells c1 (NFATc1), which is a master regulator of osteoclast differentiation. NFATc1 stimulates the terminal differentiation, survival, and osteolytic resorption of active TRAP-expressing and cathepsin K-producing osteoclasts [13,14,15,16]. Therefore, the differentiation and activity of osteoclasts during abnormal bone resorption serve as targets for treating osteoporosis and other osteolytic diseases. Certain medications used for treating osteoporosis act by preventing bone loss, such as bisphosphonates, raloxifene, calcitonin, and denosumab, which is a monoclonal antibody inhibitor of RANKL that suppresses osteoclast development; however, these medications have limitations and adverse effects [17]. Thus, a better understanding of osteoclast activation during osteoporosis can guide the development of more effective treatments with fewer adverse effects.

Neddylation, a process similar to ubiquitination, is a reversible post-translational modification of the ubiquitin-like protein, neural precursor cell-expressed developmentally downregulated protein 8 (NEDD8), on specific lysine residues of protein substrates. Neddylation is mediated by distinct sequential enzymatic steps. First, NEDD8 is activated by E1, a NEDD8-activating enzyme (NAE), which is a heterodimeric protein comprising the regulatory amyloid beta precursor protein-binding protein 1 (APPBP1) and catalytic UBA3 subunits [18]. Next, the activated NEDD8 is transferred to the NEDD8-conjugating enzyme E2, Ubc12, and the NEDD8 ligase E3 then transfers NEDD8 from E2 to the targeted substrates [19]. The most well-studied neddylation substrates are cullins [20], which are the scaffolding subunits of Cullin-RING E3 ligases (CRLs), the largest subfamily of E3 ubiquitin ligases. The NAE-mediated covalent conjugation of NEDD8 (known as neddylation) on specific lysine residues of cullins induces conformational changes in the cullins, which is required for the holoenzyme activity of CRLs for promoting protein ubiquitination and degradation of various cell-function regulators associated with several physiological and pathological process [21]. In addition to cullins, neddylation also occurs in other target proteins such as p53 [22,23] and epidermal growth factor receptor [24], which mainly causes functional changes in proteins rather than their degradation. Thus, neddylation not only is an upstream regulator of CRLs for regulating protein ubiquitination and protein degradation kinetics but also alters the subcellular localization, protein stability, and activity of proteins [19,25], It regulates numerous biological functions and is a potential therapeutic target for diseases [26,27]. 

MLN4924, a first-in-class NAE inhibitor, is a promising anticancer drug [26,28]. It blocks NAE-mediated neddylation by forming NEDD8–MLN4924 adducts, which meaningfully reduces neddylation levels of cullins and other protein substrates and results in the accumulation of CRL substrates, causing DNA damage, cell cycle arrest, apoptosis, and senescence [26,29,30,31]. However, whether neddylation is involved in the dysregulation of osteoclasts during osteoporosis and, accordingly, whether the inhibition of MLN4924-mediated neddylation can help treat osteoporosis and osteolytic diseases remain unclear. In the present study, we investigated the role and therapeutic potential of sRANKL-activated neddylation during osteoclast differentiation and osteoporosis both in vitro and in vivo.

## 2. Materials and Methods

### 2.1. Reagents

MLN4924 (Pevonedistat) was obtained from Active Biochem (Maplewood, NJ, USA). Recombinant mouse sRANKL (GFM4) was obtained from Cell Guidance Systems (Carlsbad, CA, USA). Antibodies against TRAF6 (H274), NFATc-1 (7A6), APPBP1 (H-187), NEDD8 (V-15), UBC12 (D-4), UBA3 (E-5), α-tubulin (B-7), and β-actin (C4) were acquired from Santa Cruz Biotechnology (Santa Cruz, CA, USA). Antibodies against phospho-ERK1/2 (pERK; 4370), ERK1/2 (4695), phospho-JNK (pJNK; 4668), JNK (9252), phospho-p38 MAPK (pp38; 4631), and p38-MAPK (p38; 9212) were purchased from Cell Signaling Technology (Beverly, MA, USA). Anti-GAPDH antibody (GTX627408) was purchased from GeneTex (Irvine, CA, USA). Antiubiquitin (linkage-specific K63) (ab179434) and Cullin-5 (ab184177) antibodies were purchased from Abcam (Cambridge, MA, USA).

### 2.2. Cell Cultures

The murine preosteoclastic monocytic/macrophage cell line RAW 264.7 was obtained from the Bioresources Collection and Research Center (BCRC, Hsinchu, Taiwan), which was grown in high-glucose Dulbecco’s Modified Eagle’s Medium (Gibco BRL, Gaithersburg, MD, USA) containing 10% fetal bovine serum (Gibco) and supplemented with 1x GlutaMax (Gibco) in 5% CO_2_ [32]. Mouse bone-marrow-derived macrophages (BMMs) were isolated for preparing preosteoclast cells (pre-OCs), as described previously [33]. In brief, BMM cells were cultured with 10 ng/mL M-CSF (R&D Systems, Minneapolis, MN, USA) for 3 days to obtain pre-OCs of the monocyte/macrophage lineage [34].

### 2.3. Analysis of sRANKL-Activated Neddylation Pathway

RAW264.7 cells were seeded and cultured on a 6 cm dish. After a 24 h culture, the cells were treated with various concentrations of sRANKL for 3 days or 50 ng/mL sRANKL for various time intervals. After treatments, the cells were harvested, washed, and lysed with cell lysis buffer (Cell Signaling Technology) on ice. Furthermore, the protein concentration of supernatants from cell lysates were determined using a bicinchoninic acid protein assay kit (Thermo Fisher Scientific, Waltham, MA, USA), and the equal quantities of each sample were resolved through sodium dodecyl sulfate–polyacrylamide gel electrophoresis and were then subjected to Western blotting by using specific antibodies against NEDD8, UBA3, APPBP1, UBC12, and GAPDH.

### 2.4. Cell Viability Assay

Cell viability was determined using the cell counting kit-8 (CCK-8, Sigma-Aldrich, Saint Louis, MO, USA, cat. No. 96992), as described previously [35]. The cells were seeded in a 96-well plate (5000 cells/well) and cultured in 200 µL of growth medium at 37 °C and 5% CO_2_ overnight. Next day, the medium in each well was replaced with 100 µL of cell growth medium containing sRANKL and MLN4924 for 24 or 48 h. At the indicated day, the cells were washed with phosphate-buffered saline (PBS) 3 times. Next, 10 µL of CCK-8 dye and 100 µL of α-MEM cell culture medium were added to each well, and the cells were incubated for another 1 h at 37 °C.

### 2.5. UBA3 Knockdown

RAW 264.7 cells were seeded on wells and transfected with three specific UBA3 or scrambled siRNAs (GenePharma, Shanghai, China), including UBA3 siRNA 1 (UBA3-110; sense: 5′-GGAGCCAAUGGCUGUUGAUTT-3′, antisense: 5′-AUCAACAGCCAUUGGCUCCTT-3′), UBA3 siRNA 2 (UBA3-574; sense: 5′-GGAUCAAUGGAAUGCUGAUTT-3′, antisense: 5′-AUCAGCAUUCCAUUGAUCCTT-3′), UBA3 siRNA 3′ (UBA3-627; sense: 5′-CCAAGCUCCAUUGUACCUUTT-3′, antisense: 5′- AAGGUACAAUGGAGCUUGGTT-3′), and scrambled siRNA (sense: 5′- UUCUCCGAACGUGUCACGUTT-3’, antisense: 5′-ACGUGACACGUUCGGAGAATT-3’).

### 2.6. Osteoclast Differentiation Assay

For in vitro osteoclast differentiation, RAW 264.7 cells were cultured in a medium containing various concentrations of sRANKL, with or without various concentrations of MLN4924, for various time intervals. The culture medium was replaced every 2 days. At the end of the culture period, the cells were washed, fixed with 4% paraformaldehyde, and stained with TRAP, a marker of osteoclasts, by using a TRAP staining kit (KAMIYA Biomedical Company, Seattle, WA, USA), in accordance with the instructions of the manufacturer. TRAP-positive cells with more than three nuclei were counted as osteoclasts under a phase-contrast microscope (Nikon Ti2; Melville, NY, USA).

### 2.7. Cathepsin K Activity and Pit Assays

RAW 264.7 cells were seeded on an Osteo Surface Assay 96-well plate (Corning, Kennebunk, ME, USA). After 24 h, the cells were treated with sRANKL (50 ng/mL), with or without various concentrations of MLN4924. After culturing for 7 days, the conditioned media were collected to analyze cathepsin K activity using the Cathepsin K Activity Fluorometric Assay Kit (Biovision, Milpitas, CA, USA), in accordance with the instructions of the manufacturer. Subsequently, the bone matrix resorption area on Corning Osteo surface was stained with 1% toluidine blue, visualized with a microscope (100× magnification), and quantified using Image J software (National Institutes of Health, Bethesda, MD, USA).

### 2.8. Analysis of sRANKL-Activated Downstream Signaling

RAW264.7 cells were seeded and cultured on 6 cm dishes for 24 h. Next, the cells were starved in serum-free medium and then treated with sRANKL (50 ng/mL), with or without various concentrations of MLN4924, for various time intervals. The cell lysates were then harvested using cell lysis buffer (Cell Signaling Technology). Furthermore, equal quantities of each sample were loaded into the sodium dodecyl sulfate–polyacrylamide gel electrophoresis for protein separation and were then subjected to Western blotting by using the indicated antibodies.

### 2.9. Immunoprecipitation and Western Blotting

RAW264.7 cells were starved in serum-free medium for 6 h and then treated with the indicated prescriptions. The cells were then washed with cold PBS and lysed in RIPA buffer with protease inhibitor cocktail (cOmplete; Roche, Basel, Switzerland). Cell lysates were precleared with 30 µL of protein G-agarose (EMD Millipore, Bedford, MA, USA), and the precleared lysates were incubated with the TRAF6 antibody at 4 °C for 18 h. Next, protein G-agarose beads were added to each lysate and incubated 4 °C for 2 h. Subsequently, the beads were washed three times with lysis buffer, and the bound proteins were separated using SDS-PAGE followed by Western blotting with the specific antibodies.

### 2.10. sRANKL Combined Ovariectomy-Induced Osteoporosis Mouse Model

To assay osteoporosis in vivo, 8-week-old female C57BL/6J mice (BioLASCO, Taipei, Taiwan) were used with the approval of Institutional Animal Care and Use Committee of Chang Gung Memorial Hospital (Chaiyi, Taiwan) (IACUC number: 2013100102). To evaluate the role of neddylation inhibition in osteoporosis in vivo in a short period, a combination of sRANKL injections and ovariectomy mouse model was established, as described in a previous study [36]. The mice were administered general anesthesia and subjected to either a sham operation or bilateral ovariectomy (OVX) (*n* = 6 per group). We randomly divided the mice into four groups: sham (sham operation with saline injection), OVX (OVX with saline injection), OVX and sRANKL (OVX with sRANKL injection), and OVX and sRANKL and MLN (OVX with sRANKL and MLN4924 injections). Then, 48 h after OVX, saline or sRANKL (1 mg/kg) was injected intraperitoneally into the mice once daily for 2 days, followed by intraperitoneal administration of MLN4924 (10 mg/kg) or saline once daily for 2 weeks. Eventually, the mice were killed by CO_2_ asphyxiation, and the serum was collected for the evaluation of Cross-linked C-telopeptide of type I collagen (CTX-1) (CEA665Mu) by using specific ELISA kits (USCN life science incorporation, Wuhan, Hubei, China). Mouse tibias and femurs were harvested. The tibias were fixed with 4% paraformaldehyde for micro-CT and histological analysis, and the femurs were subjected to a bending test using MTS and tomographic analysis using micro-CT.

### 2.11. Micro-CT Scanning

The fixed tibias (*n* = 6 per group) were analyzed using a high-resolution micro-CT scanner (Skyscan 1176; Skyscan, Aartselaar, Belgium), with the following settings: isometric resolution at 9 mm and X-ray energy settings of 80 kV and 80 mA. Bone volume/tissue volume, trabecular separation, trabecular thickness, and trabecular number were measured using the resident reconstruction program (DataViewer, Ver.1.5.0.0, Aartselaar, Belgium).

### 2.12. Biomechanical Testing

The femurs (*n* = 12 per group) were performed on an MTS Synergie 200 (Testresources, Shakopee, MN, USA) with three-point bending test to failure. The applied speed of load was 1 mm/min, settling time of 30 s, and distance between the points of 10 mm with a preload of 1 N, load cell of 500 N. The structural properties, including modulus, load at break, stress at yield, and strain at yield were determined by the force–displacement plot.

### 2.13. Histological Analysis

The isolated tibias (*n* = 6 per group) were subjected to undecalcified frozen sectioning using Kawamoto’s film method, as described previously [37]. Finally, the frozen sections were stained with hematoxylin and eosin and TRAP; the images were captured with the Olympus BX51 microscope system (Olympus), and the bone histomorphometry parameters were evaluated with DP2-BSW software, as described by Dempster et al. [38]. The sections were measured within an area of 350–600 µm distal to the growth plate. Bone resorption parameters, including number of osteoclasts (multiple-nucleus with TRAP-positive) per 0.1 mm of bone length, osteoclast surface/bone surface, and erosion surface/bone surface, were also determined.

### 2.14. Osteogenic Differentiation and Alizarin Red S Stain

The osteoblastic cell line, MC3T3-E1, was purchased from BCRC (Hsinchu, Taiwan) and was grown in αMEM supplemented with 10% FBS without ribonucleosides and deoxyribonucleosides at 37 °C. For osteogenic differentiation, MC3T3-E1 cells were cultured in growth medium supplemented with L-ascorbic acid (50 µg/mL; Sigma-Aldrich) and β–glycerophosphate disodium salt hydrate (10 mM; Sigma-Aldrich) for 20 days. The differentiation medium was refreshed every 3–4 days. After differentiation, the calcium deposits were stained and quantified using the Alizarin Red S Staining Quantification Assay Kit (ScienCell Research Laboratories, Carlsbad, CA, USA).

### 2.15. Statistical Analyses

Data are presented as the mean ± standard deviation. Between-group differences were compared with one-way ANOVA, and *p* < 0.05 was considered significant. All statistical analyses were performed using Prism (version 6.0, GraphPad Software, CA, USA).

## 3. Results

### 3.1. Neddylation Pathway Is Upregulated in sRANKL-Mediated Osteoclastogenesis

RANK is a member of the TNF receptor family expressed by osteoclasts and their precursors. The interaction of RANK with its soluble ligand (i.e., sRANKL) through TRAF6 has been identified as the critical pathway to control bone resorption [39]. Furthermore, neddylation critically governs TRAF6-mediated inflammation. Furthermore, the ubiquitin-like NEDD8 activity is stimulated by sRANKL [40]. Therefore, to deduce the association between NEDD8 modification and osteoporosis, we examined sRANKL-mediated osteoclastic differentiation. However, whether the neddylation pathway is involved in sRANKL-mediated osteoclast differentiation remains unclear.

**Figure 1:**
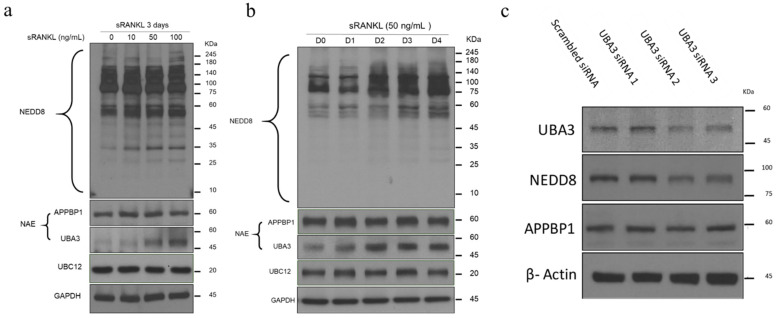

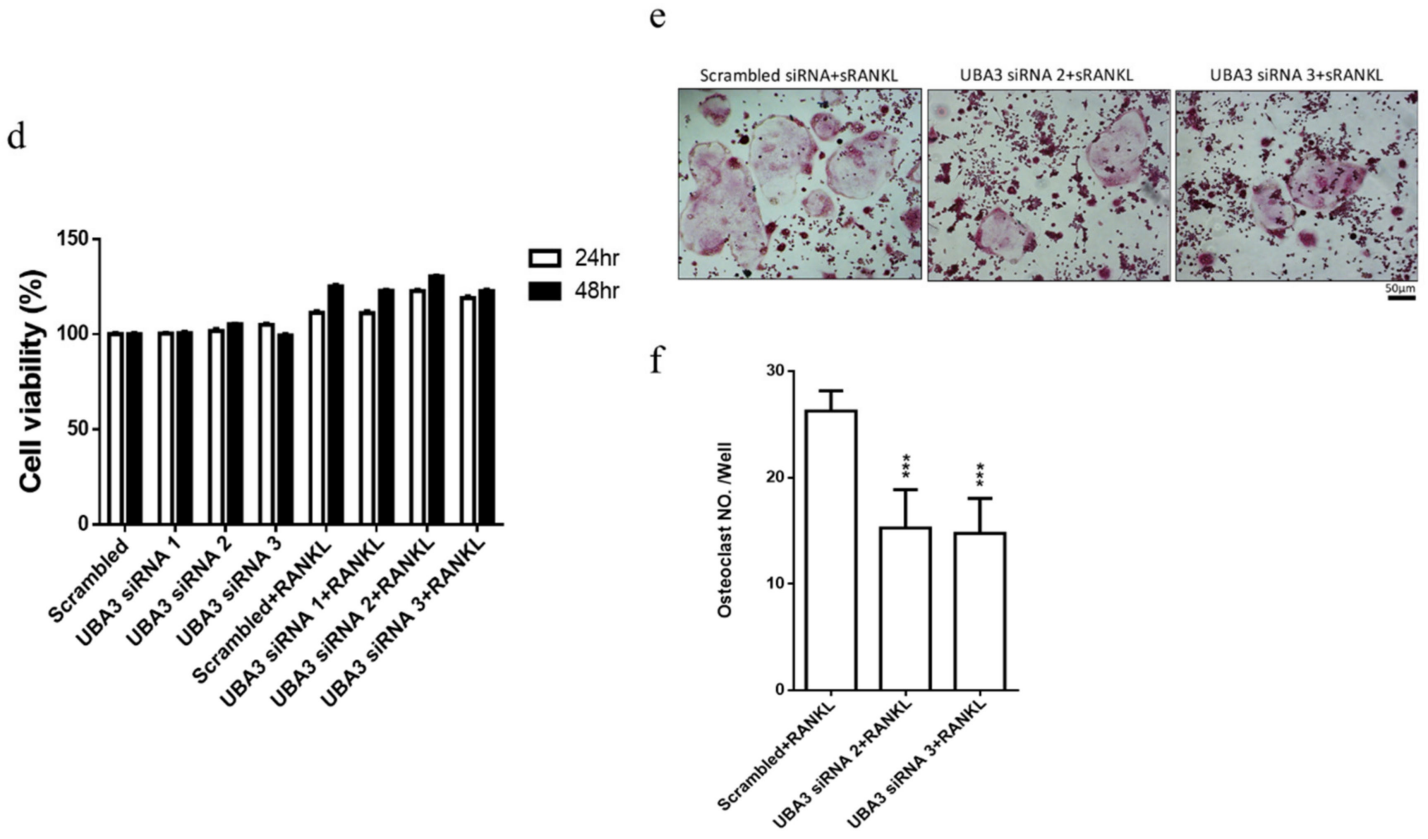
Effect of soluble receptor activator of nuclear factor-κB ligand (sRANKL) on the neddylation pathway and the effect of UBA3 siRNA on macrophages during osteoclast differentiation in vitro. RAW264.7 cells were seeded on wells and were treated with various concentrations of sRANKL (**a**) or 50 ng/mL sRANKL at the indicated day (**b**). The cells were lysed and subjected to Western blotting by indicated antibodies in the neddylation pathway. RAW264.7 cells were transfected with three UBA3-specific or scrambled siRNA and were harvested, lysed, and further analyzed through Western blotting for assessing the expression of UBA3, NEDD8, and APPBP1 (**c**), and the cell viability was tested (**d**). The UBA3 siRNA 2, UBA3 siRNA 3, or scrambled siRNA transfected RAW264.7 cells were further treated with sRANKL (50 ng/mL), and the TRAP-stained positive giant osteoclasts (more than three nuclei) were photographed (**e**) and further counted (**f**). *** *p* < 0.05.

First, the stimulatory effect of sRANKL on neddylation in RAW 264.7 cells was determined. The results revealed that NEDD8 modification of proteins was enhanced dose-dependently by sRANKL after 3-day incubation (Figure 1a) as well as time-dependently by 50 ng/mL sRANKL (Figure 1b). Furthermore, sRANKL significantly increased the expression of the catalytic subunit UBA3 but not of the regulatory subunit APPBP1 during osteoclast differentiation, indicating a key role of UBA3 in sRANKL-mediated osteoclastic differentiation.

### 3.2. UBA3 Knockdown Attenuates Osteoclast Differentiation in RAW 264.7 Cells

To determine the functional effect of UBA3 in sRANKL-activated osteoclastic differentiation, three specific RNAi molecules by targeting UBA3 mRNA were transfected into RAW 264.7, for knocking down UBA3 expression. As illustrated in Figure 1c, UBA3 expression level was significantly reduced in RAW 264.7 cells after transfection with siRNA 2 and 3 compared with scrambled control and siRNA 1; no siRNA had a significant effect on RAW 264.7 cell viability, irrespective of sRANKL treatment (Figure 1d). The UBA3 knockdown RAW 264.7 cells were treated with sRANKL to induce osteoclastic differentiation. The TRAP staining data indicated that scrambled control and siRNA 1 did not affect sRANKL-stimulated osteoclastic differentiation, whereas siRNA 2 and 3 significantly reduced the number of osteoclasts (Figure 1e,f). These data demonstrated that the siRNA specific for UBA3 inhibits osteoclast differentiation, indicating that UBA3 expression and activity in NAE-mediated neddylation are essential during sRANKL-stimulated osteoclastic differentiation.

### 3.3. Inhibition of Neddylation Significantly Suppresses Osteoclast Differentiation in sRANKL-Stimulated Macrophages

**Figure 2:**
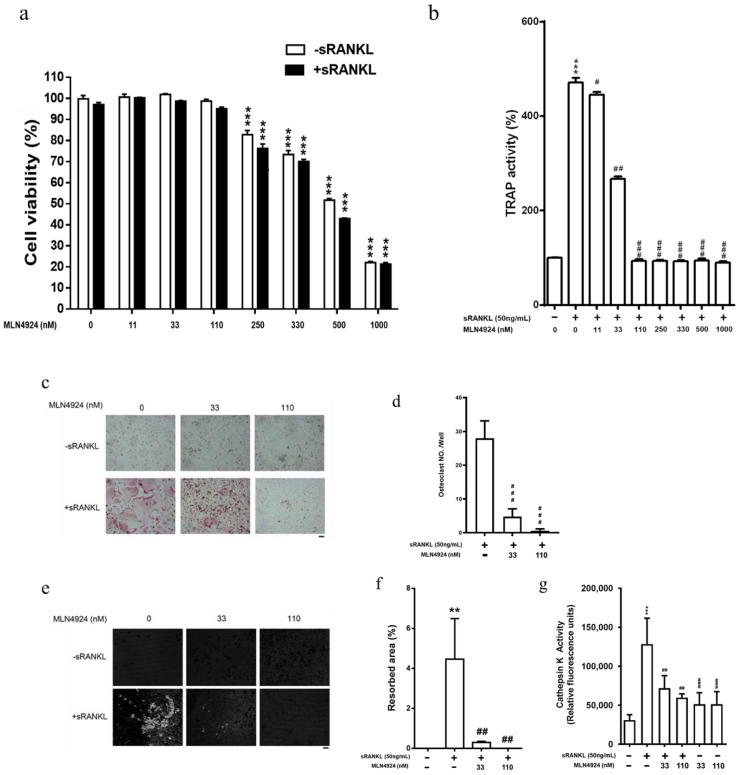
Effect of MLN4924 on proliferation and osteoclastogenesis in soluble receptor activator of nuclear factor-κB ligand (sRANKL)-stimulated RAW 264.7 cells. Cells were seeded into 96-well plates, cultured with sRANKL (100 ng/mL), and indicated the concentration of MLN 4924 for 7 days. Cell proliferation assay at 48 h (**a**), *** compared with 0 nM *p* < 0.001. The TRAP activity was also measured (**b**), *** compared with control *p* < 0.001, # compared with sRANKL, *p* < 0.05; ## compared with sRANKL, *p* < 0.01; ### compared with sRANKL, *p* < 0.001. In 24-well plate, osteoclasts were identified through TRAP staining (**c**). The TRAP-stained positive giant osteoclasts were quantified (**d**), ### compared with sRANKL, *p* < 0.001. Cells were removed from the Corning Osteo Surface Assay 96-well plate and visualized with 1% toluidine blue (**e**). All photomicrographs were captured using an inverted microscope at a magnification of 100×. Quantification of the resorbed area (**f**), ** compared with control *p* < 0.01; ## compared with sRANKL, *p* < 0.01. Cells treated with indicated groups were harvested and subjected to detection of cathepsin K activity (**g**). Scale bar represents 100 μm. *** compared with control *p* < 0.001, ## compared with sRANKL, *p* < 0.01; ### compared with sRANKL, *p* < 0.001.

**Figure 3:**
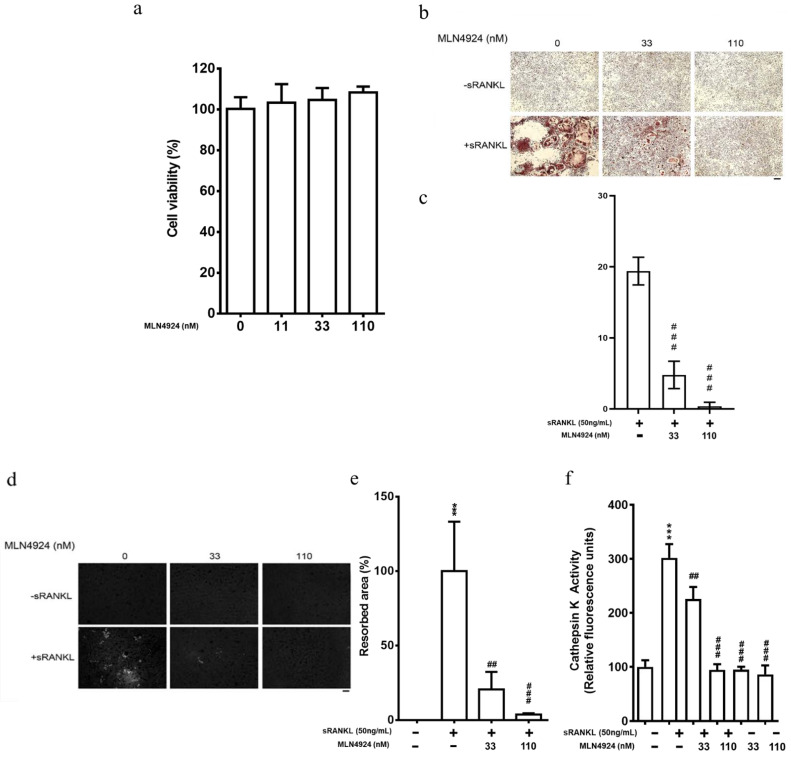
MLN4924 attenuates soluble receptor activator of nuclear factor-κB ligand (sRANKL)-induced osteoclast differentiation derived from bone-marrow-derived macrophage (BMM) in vitro. BMMs were seeded into 96-well plates, and cultured with sRANKL (100 ng/mL) and M-CSF (40 ng/mL), and indicated the concentration of MLN4924 for 7 days. (**a**) The viability of BMMs was determined using the CCK-8 assay after treatment with M-CSF and various concentrations of MLN4924 for 7 days. In a 24-well plate, osteoclasts were identified through TRAP staining (**b**). The TRAP-stained positive giant osteoclasts were quantified (**c**). The cells were removed from the Corning Osteo Surface 96-well plate and visualized with 1% toluidine blue (**d**). All photographs were captured using an inverted microscope at a magnification of 100×. Quantification of the resorbed area (**e**). Treated cells were harvested and subjected to cathepsin K activity detection (**f**). Scale bar represents 100 μm. *** compared with control *p* < 0.001, ## compared with sRANKL, *p* < 0.01; ### compared with sRANKL, *p* < 0.001.

Our data revealed that the UBA3 subunit knockdown attenuated osteoclastic differentiation. A study demonstrated that the persistent and severe inactivation of neddylation by MLN4924 triggers apoptotic events in RAW 264.7 macrophages [41]. However, the effect of MLN4924-mediated inhibition of neddylation in sRANKL-stimulated osteoclastic differentiation in macrophages has been unclear. We observed that higher doses (250–1000 nM) of MLN4924, but not lower doses (11–110 nM), significantly suppressed macrophage proliferation, regardless of sRANKL treatment (Figure 2a). Moreover, higher doses of MLN4924, but not lower doses, significantly reduced TRAP activity in RAW 264.7 cells treated with sRANKL (Figure 2b). Next, we evaluated the effect of lower doses of MLN4924 on osteoclast differentiation and activities in RAW 264.7 cells. As illustrated in Figure 2c, lower doses of MLN4924 (33 and 110 nM) markedly inhibited mature osteoclast formation, and the numbers of osteoclasts were quantified (Figure 2d). The pit assay for assessing osteoclast activity also indicated that low doses of MLN4924 inhibited the area of resorption on the bone disc (Figure 2e,f). MLN4924 (33 and 110 nM) also inhibited cathepsin K activity, an indicator of bone resorption (Figure 2g). The same attenuation effects were noted in bone-marrow-derived macrophages (Figure 3a–f). Together, these data indicated that the inhibition of neddylation by MLN4924 significantly suppressed sRANKL-mediated osteoclastogenesis in vitro. However, whether the reduced activities of the osteoclasts by MLN4924 treatment are largely dependent on the reduced number of osteoclasts or not.

### 3.4. Inhibition of Neddylation by MLN4924 Diminishes sRANKL-Activated Downstream Signaling

**Figure 4:**
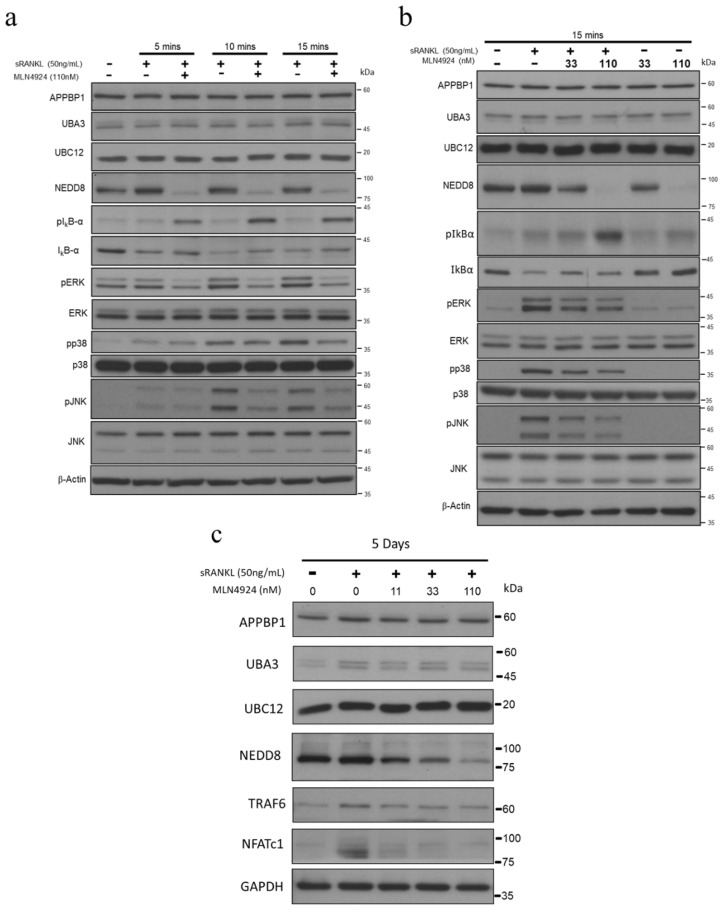
Effect of MLN4924 on neddylation and sRANKL-stimulated signaling pathway in RAW 264.7 cells (**a**) RAW 264.7 cell were pretreated with 110 nM MLN4924 for 30 min, followed by treatment with sRANKL (50 ng/mL) at 5, 10, and 15 min, or (**b**) 33 and 110 nM MLN4924 for 30 min, followed by treatment with sRANKL (50 ng/mL) at 15 min. Cell lysates were collected and subjected to immunoblotting analysis for neddylation (APPBP1, UBA3, UBC12, and NEDD8) and sRANKL-induced pathway (phosphorylation of I_k_B-α (pI_k_B-α), ERK (pERK), P38 (pP38), and JNK (pJNK)). (**c**) RAW 264.7 cells were cotreated with sRANKL (50 ng/mL) and various concentrations of MLN4924 (11–110 nM) for 5 days. Cell lysates were collected and subjected to Western blotting with neddylation-related proteins (APPBP1, UBA3, UBC12, and NEDD8) and specific antibodies for sRANKL-induced proteins (TRAF6 and NFATc1).

Since our data revealed that MLN4924 attenuates sRANKL-mediated osteoclastogenesis in vitro, we further investigated the mechanisms underlying sRANKL-induced downstream signaling associated with the neddylation pathway (Figure 4a). Initially, we observed that NEDD8 modification was increased in RAW 264.7 cells after sRANKL treatment at 5, 10, and 15 min. Additionally, UBA3 expression was enhanced. These results are consistent with those presented in Figure 1a,b. MLN4924 treatment decreased both these upregulated levels but did not significantly affect the level of APPBP1.

The binding of sRANKL recruits TNF-receptor-associated cytoplasmic factors to the cytoplasmic domain of RANK, thereby activating the ERK, p38-MAPK, and JNK signaling pathways during osteoclastogenesis [11,42,43]. Hence, the downstream molecules—levels of TRAF6 and phosphorylated ERK, p38-MAPK, and JNK—were analyzed simultaneously. The data revealed that TRAF6 protein levels were slightly increased and that phosphorylation of ERK, p38-MAPK, and JNK was increased at 5, 10, and 15 min after sRANKL stimulation. However, these phenomena were significantly repressed from 10 min in the MLN4924-treated group (Figure 4a), and the effect was stronger with 110 nM MLN4924 than with 33 nM MLN4924 (Figure 4b).

NFATc-1 is the ultimate target of RANKL-activated RANL signal cascades and a key transcription factor for osteoclast differentiation. Its ectopic expression in preosteoclast cells causes their differentiation into osteoclasts in the absence of sRANKL [13]. Hence, we investigated the effect of MLN4924 on NFATc-1 levels. As presented in Figure 4c, increased NFATc-1 levels induced by various concentrations of sRANKL were significantly suppressed by MLN4924 treatment after 5 days. Notably, UBA3 was also apparently suppressed by MLN4924.

**Figure 5:**
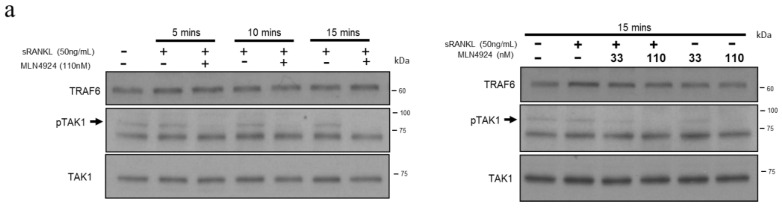

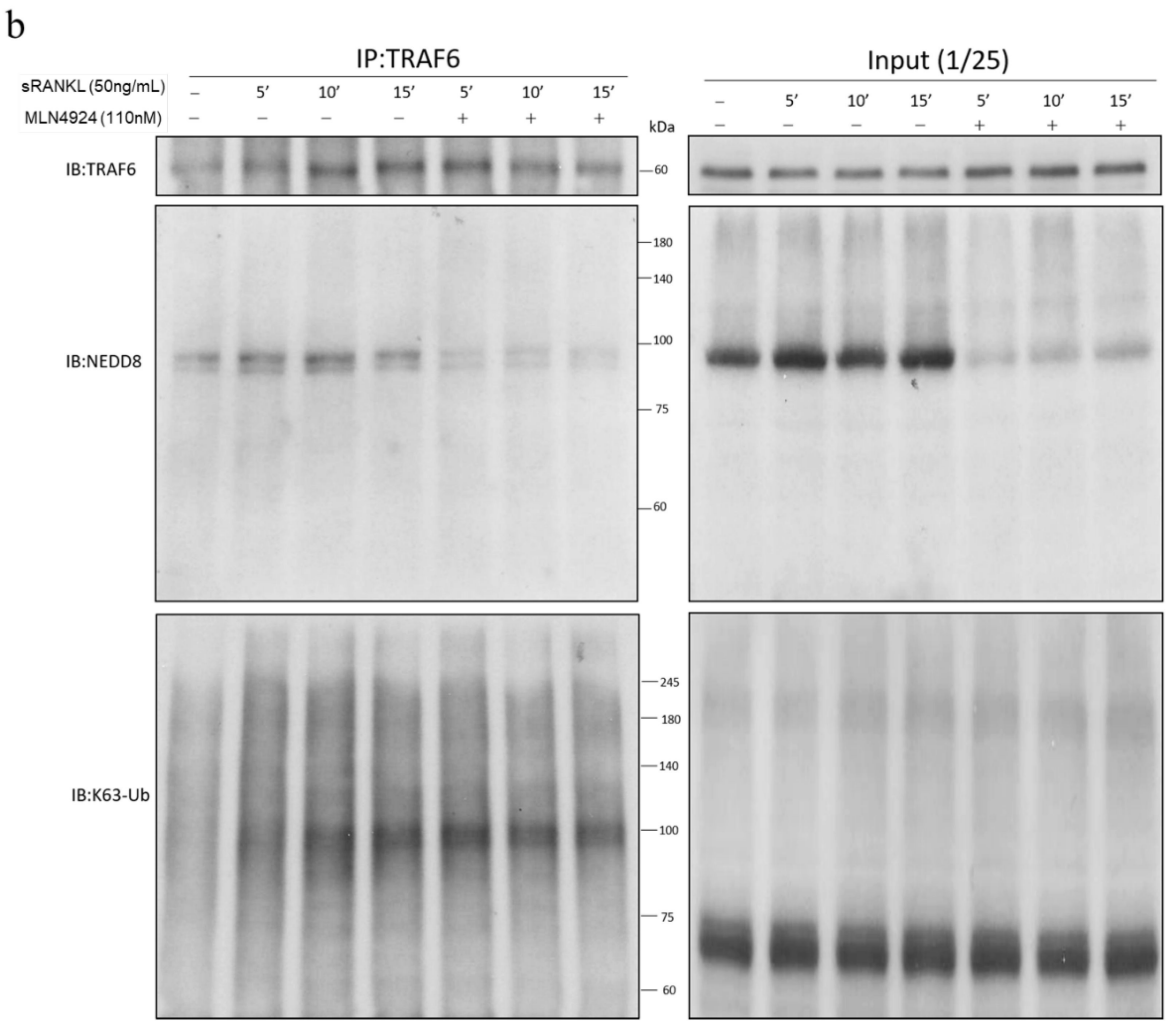
MLN4924 inhibits the soluble receptor activator of nuclear factor-κB ligand (sRANKL)-induced phosphorylation of TAK1 to reduce the K63-Ub level of TRAF6. (**a**) RAW 264.7 cells were pretreated with 110 nM MLN4924 for 30 min, followed by treatment with sRANKL (50 ng/mL) at 5, 10, and 15 min, or with 33 and 110 nM MLN4924 for 30 min, followed by treatment with sRANKL (50 ng/mL) at 15 min. Cell lysates were collected and subjected to Western blotting with TRAF6 and phosphorylation of TAK1 (pTAK1). (**b**) The cell lysates pretreated with 110 nM MLN4924 for 30 min, followed by treatment with sRANKL (50 ng/mL) at 5, 10, and 15 min, were subjected to immunoprecipitation (Co-IP). Total proteins were immunoprecipitated with anti-TRAF6 and immunoblotted with anti-NEDD8, anti-K63-Ub antibodies. Right panel input represents 1/25 of cell lysates used in the Co-IP.

For the upstream signal in the RANKL pathway, RANKL stimulation activates endogenous TAK1 activity after TRAF6 polyubiquitination [44]. The formation of the TRAF6–TAK1 complex with RANK is vital for RANK signaling (Figure 5a). After sRANKL stimulation, TAK1 phosphorylation was enhanced but was inhibited by MLN4924 50 nM at 5 min and 110 nM at 15 min. Therefore, we hypothesized that MLN4924 plays a role early in the RANKL pathway, so its effect on TRAF6 must be elucidated.

Lysine (K) 63-linked polyubiquitination on TRAF6 is a critical upstream signal for RANKL-induced osteoclast differentiation [40]. Therefore, we examined whether MLN4924 inhibits RANKL-activated K63-linked polyubiquitination of TRAF6 (K63-Ub) by using CO-IP. The results revealed that the K63-Ub was time-dependently upregulated after RANKL stimulation (Figure 5b). By contrast, after MLN4924 treatment, K63-Ub was diminished at the various RANKL-treated time points. Together, these data indicated that MLN4924 strongly inhibits osteoclast formation by decreasing K63-Ub of TRAF6, which then inactivates the ERK, p38-MAPK, and JNK pathways and, thus, inhibits the downstream transcription factor NFATc-1.

### 3.5. Assessment of MLN4924 Treatment on OVX/sRANKL-Mediated Bone Loss In Vivo and Biomechanical Properties

**Figure 6:**
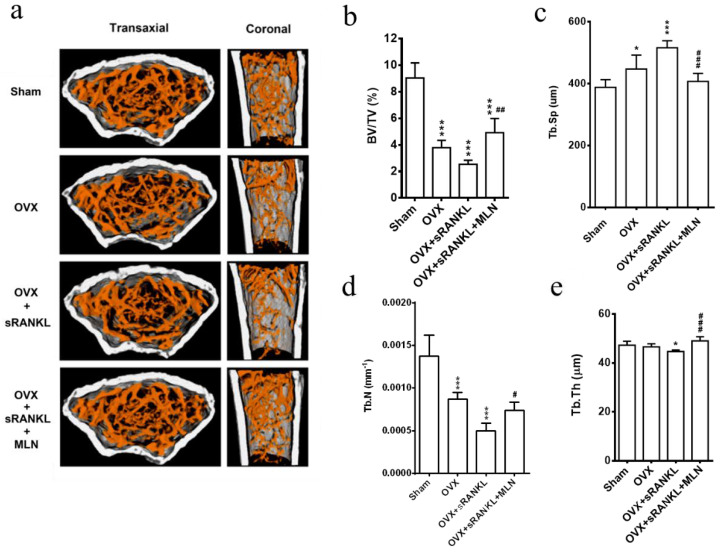
Assessment of MLN4924-treatment in ovariectomy (OVX) and soluble receptor activator of nuclear factor-κB ligand (sRANKL)-induced osteoporosis mouse model. (**a**) Micro-computed tomography (micro-CT) three-dimensional images of the distal femurs of mice with indicated treatments; the transaxial plane is presented in left panels, and the coronal plane is presented in right panels. (**b**–**e**): micro-CT measurement of trabecular bone parameters, including bone volume/total volume (**b**), trabecular space (**c**), trabecular number (**d**), and trabecular thickness (**e**). Values are expressed as mean ± SD, N = 6 per group. * OVX and OVX and sRANKL compared with sham, *p* < 0.05; *** OVX and OVX and sRANKL compared with sham, *p* < 0.001, # compared with sRANKL, *p* < 0.05; ## compared with OVX and sRANKL, *p* < 0.01; ### compared with OVX and sRANKL, *p* < 0.001.

Since MLN4924 inhibits the sRANKL-induced osteoclastogenesis in vitro, its therapeutic potential against osteoporosis was investigated using an OVX and sRANKL-mediated osteoporosis in mice [36,45]. In our in vivo experiments, μCT analysis revealed severe trabecular bone loss at the proximal tibial metaphysis (Figure 6a), along with a significant decrease in trabecular bone volume (Figure 6b) and number (Figure 6c) and an increase in trabecular separation (Figure 6d) in the OVX and sRANKL group compared with the sham group. Notably, OVX- and sRANKL-mediated phenomena were significantly inhibited in the OVX and sRANKL and MLN group.

**Figure 7:**
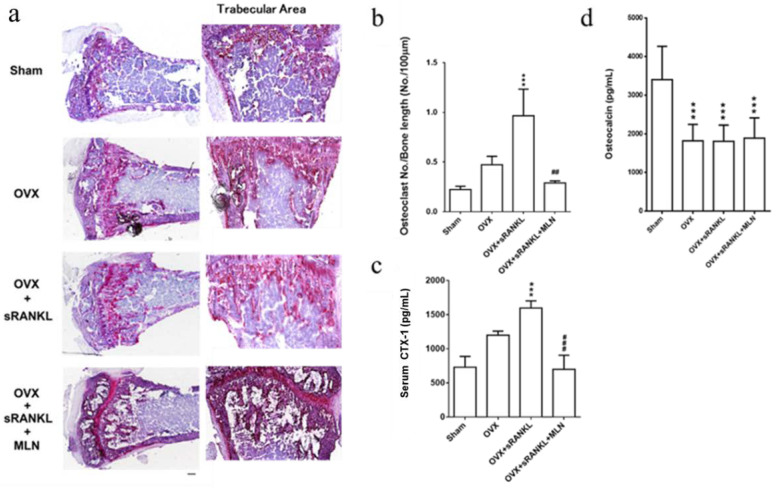
Histologic and immunohistochemical studies of the uncalcified bone section of the proximal tibia. (**a**) Hematoxylin- and TRAP-stained sections of tibial metaphyses from sham, ovariectomy (OVX), OVX and soluble receptor activator of nuclear factor-κB ligand (sRANKL), and OVX and sRANKL and MLN4924 (MLN) mice. (**b**) Osteoclast number per bone length. (**c**) The serum level of CTX-1 and osteocalcin (**d**) was measured using specific ELISA in the Sham, OVX, OVX and sRANKL, and OVX and sRANKL and MLN groups. Values are the mean ± S.D (6 mice per group). *** OVX and OVX and sRANKL compared with sham, *p* < 0.001; ## compared with OVX and sRANKL, *p* < 0.01; ### compared with OVX and sRANKL, *p* < 0.001.

We further analyzed the histologic and immunohistologic tissue sections of the tibial bone of all groups (*n* = 6). TRAP staining analysis revealed a profound increase in bone density (pink color) in the OVX and sRANKL and MLN group compared with the other groups (Figure 7A). Compared with the sham group, the osteoclasts in the OVX and OVX and sRANKL groups were more diffuse and migrated from the epiphyseal region to the trabecular bone in the metaphyseal regions. However, in the OVX and sRANKL and MLN group, few migrated osteoclasts were noted, and the bone morphology resembled that of the sham group. Consistent with this finding, the number of osteoclasts per bone length also significantly increased in the OVX and OVX and sRANKL groups, indicating elevated osteoclastogenesis, whereas it was deceased in the OVX and sRANKL and MLN group (Figure 7b). Collectively, these results clearly indicated a significant inhibition of in vivo OVX- and sRANKL-mediated osteoporosis by MLN4924.

Bone-turnover suppression has been associated with osteoclast inhibition, due to the negative feedback to osteoblasts. To further investigate the effect of MLN4924 on bone-turnover markers in vivo, we investigated the level of serum C-terminal cross-linked telopeptide of type I collagen (CTX) and osteocalcin, which are biomarkers of bone resorption and bone formation, respectively [46]. Serum CTX-1 level was notably increased in the OVX and sRANKL group but was significantly inhibited in the OVX and sRANKL and MLN group (Figure 7c); the level of serum osteocalcin remained unaffected (Figure 7d), indicating MLN4924 might affect bone resorption more than formation.

**Figure 8:**
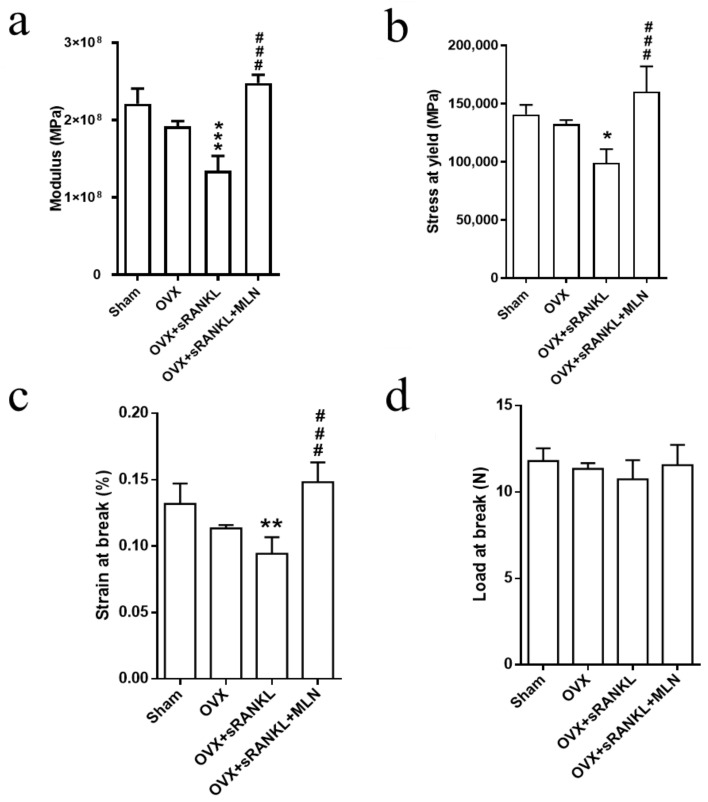
Mechanical properties of femurs in the control, sham, ovariectomy (OVX), OVX and soluble receptor activator of nuclear factor-κB ligand (sRANKL), and OVX and sRANKL and MLN4924 (MLN) groups. (**a**) Elastic modulus, (**b**) stress at yield, (**c**) strain at break, and (**d**) load at break. Values are mean ± S.D, 12 mice per group. * Compared with sham, *p* < 0.05; ** compared with sham, *p* < 0.01; *** compared with sham, *p* < 0.001; ### compared with OVX and sRANKL, *p* < 0.001.

Loss of bone architecture and mechanical strength result in bone fragility and low energy fracture. Therefore, we further examined the biomechanical property of the femurs from the OVX- and sRANKL-mediated osteoporosis model. Femur shaft fracture testing revealed that modulus, stress at yield, and strain at break were significantly reduced in the OVX and OVX and sRANKL groups compared with the sham group (Figure 8a–c, respectively). MLN4924 treatment enhanced the abovementioned parameters in the OVX and sRANKL group. Although no significant difference was noted in terms of the load at break (Figure 8d), a similar trend as that of the other parameters was noted. These results imply that MLN4924 may prevent the loss of bone strength induced by OVX- and sRANKL-mediated osteoporosis.

### 3.6. Effect of MLN4924 on Osteoblast Differentiation in MC3T3-E1 Cells

**Figure 9:**
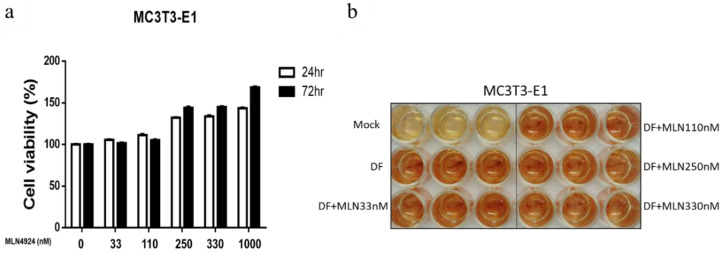

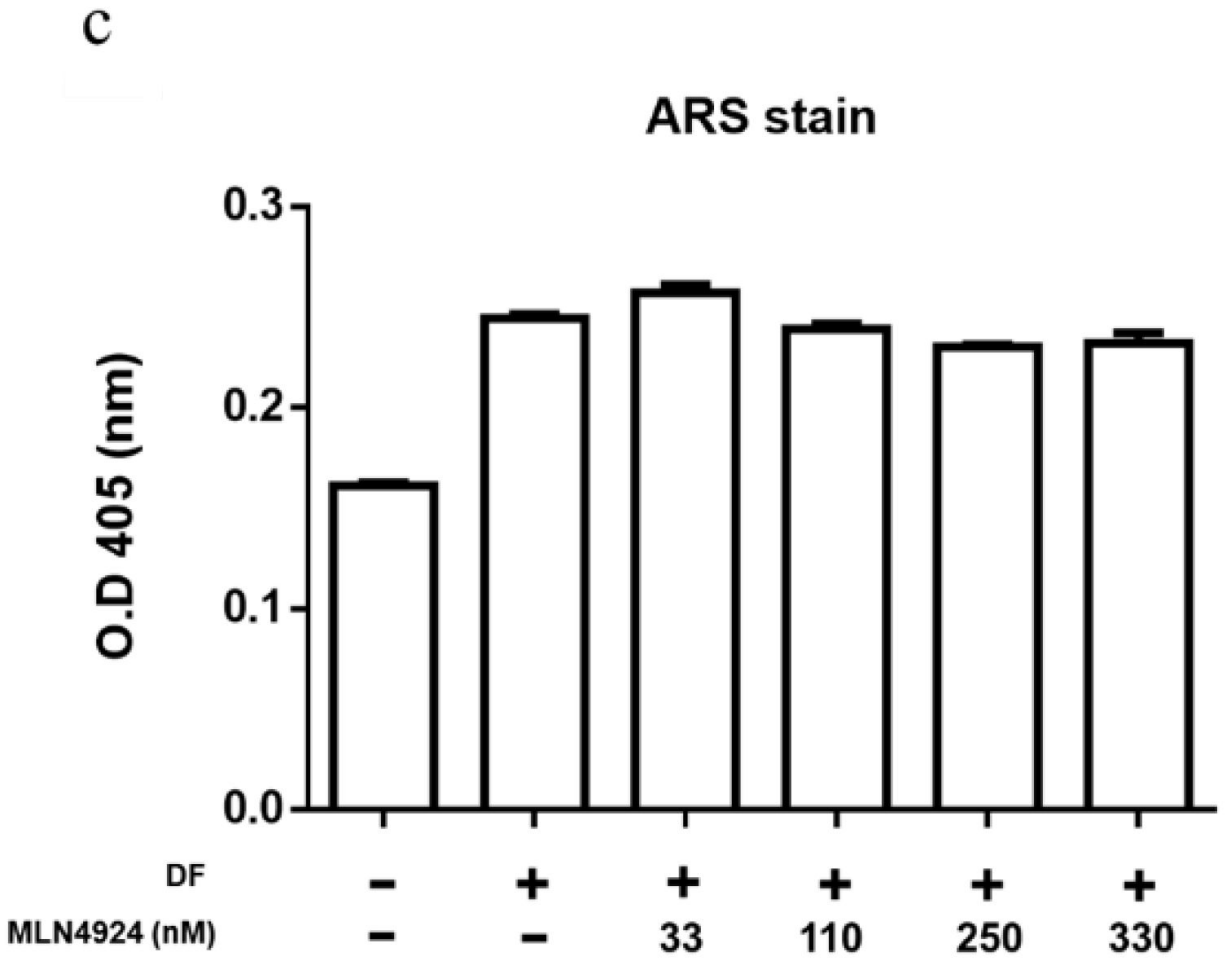
Effect of MLN4924 during osteogenesis. Mouse osteoblastic MC3T3-E1 cells were seeded in wells and incubated in osteoblast differentiation medium (DF), with or without various concentrations of MLN4924. After 21 days of differentiation, MC3T3-E1 cell viability was determined using crystal violet staining (**a**). Furthermore, the osteogenic differentiation of MC3T3-E1 cells was confirmed with Alizarin Red S staining (**b**). (**c**) The red-colored Alizarin-stained area was quantified on day 21.

Given the unchanged levels of serum osteocalcin after MLN4924 administration, we assessed the effect of MLN4924 on osteoblast differentiation in vitro by using MC3T3-E1 cells. The results revealed that fewer than 330 nM of MLN 4924 did not affect the cell viability (Figure 9a) and osteoblast differentiation in MC3T3-E1 cells (Figure 9b,c), indicating that MLN 4924 does not affect bone formation.

## 4. Discussion

Bone homeostasis is maintained by the dynamic balance between two opposing but equilibrating processes: bone-synthesizing osteoblasts and bone-degrading osteoclasts [29]. Any homeostatic imbalance results in a decrease and an increase in osteoblast and osteoclast activity, respectively. As a result, bone strength is reduced, thereby increasing the risk of fracture. RANK is a member of the TNF family expressed by osteoclasts and their precursors, and its interaction with its ligand (sRANKL) has been identified as the final common pathway through which bone resorption is regulated [36]. The RANKL/RANK signaling pathway in osteoclastogenesis offers the molecular targets for developing therapeutics to treat bone loss [47]. Although the multinucleated giant and osteoclast cells originated from similar backgrounds, RANKL stimulation is the specific factor for osteoclast formation [48]. Recent studies have documented sRANKL-induced-osteoclastic differentiation through the repressed expression of negative osteoclastogenic genes [35]. Binding of sRANKL to RANK activates receptor trimerization and recruitment of TRAF6, an essential signaling component of osteoclasts and osteoclast precursors, the activity of which is regulated by ubiquitination [37]. Ubiquitin–proteasome-mediated protein degradation plays a vital role in RANKL-mediated osteoclastogenesis. Ubiquitin-mediated RANK signaling has also been associated with the excessive resorption noted in Paget’s disease of bone [38]. In addition, the CRL E3 ubiquitin ligases, well-studied targets of neddylation, when conjugating with NEDD8 and the NEDD8-conjugated CRL E3 ligases, deliver ubiquitin residues to their target proteins by forming an E2–E3 complex, causing protein polyubiquitination [34]. However, the role of neddylation in osteoclastogenesis remains unknown. Our data indicated that the E1 subunit UBA3 of neddylation, regulating most ligases in the ubiquitin CRL family, was involved in RANKL-stimulated osteoclastogenesis (Figure 1). sRANKL-activated TRAF6 leads to the autoamplification of NFAT-c1, which promotes osteoclast differentiation [40,41]. The levels of TRAF6, phosphorylated ERK, phosphorylated p38-MAPK, phosphorylated JNK, and NFATc-1 were all suppressed by MLN4924, implying that neddylation inhibition reduced the downstream signals of the sRANKL pathway at an early stage. Furthermore, µCT analysis of the trabecular bone microstructures revealed deteriorated bone microarchitectures in the OVX/sRANKL group, which were reversed by MLN4924, indicating an inhibition of sRANKL-induced bone loss. Histological analysis revealed that MLN4924 significantly diminished the number of osteoclasts compared with the OVX and sRANKL group in vivo (Figure 7b). This therapeutic effect was supported by decreased osteoclast activities, as demonstrated by TRAP staining, and decreased levels of CTX-1, which is one of the degraded bone products of type 1 collagen, reflecting the enzymatic activity of cathepsin K [43]. Additionally, the OVX/sRANKL/MLN group demonstrated improved biomechanical properties, including modulus, load at break, stress at yield, and strain at yield. These data indicated that neddylation plays a vital role in RANKL-stimulated osteoclastogenesis.

NEDD8-activating enzyme (NAE) is a heterodimeric molecule consisting of APPBP1 and UBA3 [49]. First, NAE binds to ATP and NEDD8 and catalyzes the formation of a NEDD8–AMP intermediate. This intermediate binds to the adenylation domain of NAE. NEDD8–AMP reacts with the catalytic cysteine in UBA3, during which NEDD8 is transferred to the catalytic cysteine, resulting in a high-energy thioester linkage. NAE then binds to ATP and NEDD8 to generate a second NEDD8–AMP, forming a fully loaded NAE carrying two activated NEDD8 molecules. MLN4924 is a mechanism-based inhibitor, with enzyme inhibition occurring through the formation of a tight-binding NEDD8-MLN4924 adduct [31]. Notably, mechanistic studies have concluded that MLN4924 suppressed the expression of UBA3 but not APPBP1 at 5 days (Figure 4c). The effect on the osteoclastogenesis was similar to that achieved with UBA3 knockdown (Figure 1c,e,f). To date, no study has analyzed how the MLN4924 decreases UBA3 protein. Our data indicated that MLN4924 might decrease K63-Ub of TRAF6 to inhibit the subsequent cascades of RANKL-stimulated osteoclastogenesis; however, this should be validated by further studies.

Osteoclastogenesis can also be induced by several pathways in other disease such as chronic inflammation or infection [50]. Lipopolysaccharide (LPS) promotes inflammation related to osteoclast differentiation by activating the MAPK pathway; this concept is similar to the mechanism of sRANKL in RAW264.7 cells [51,52]. Neddylation inhibition can successfully suppress the LPS-induced cytokine upregulation [53]. Additionally, LPS-activated CRL5 interacts with TRAF6 and promotes the K63-Ub of TRAF6 to activate the subsequent cascades [54]. Our data revealed that the sRANKL-induced K63-Ub of TRAF6 was attenuated by MLN4924 treatment (Figure 5b. Thus, MLN4924-mediated inhibition of sRANKL-mediated osteoclastogenesis might downregulate K63-Ub of TRAF6 by blocking CRL5 neddylation to decrease CRL5–TRAF6 interaction; this can lead to the attenuation of the subsequent cascades. Moreover, other regulators for TRAF6 polyubiquitination including p63 and CYLD may also be involved [55]. Further research is warranted to verify these assumptions.

Proteasome inhibitors normalize bone remodeling by regulating the ubiquitin–proteasome system, which mediates the catabolism of various proteins, such as bone morphogenetic proteins [56], β-catenin [57], and zinc-finger transcription factor Gli2 [58], which regulate bone remodeling [59]. E3 ubiquitin ligases also regulate the transcription factor Runx2, which is involved in osteoblast differentiation [60]. Consistent with this hypothesis, a few studies have stated that the inhibition of proteasome reduced bone resorption and increased osteogenesis [61,62]. However, studies on the long-term treatment of these inhibitors are required to determine their toxicity and adverse effects. Bimodal interactions between preexisting estrogen-deficiency osteoporosis and bone metastasis development provided evidence supporting the differences in tumor growth and colonization between healthy individuals and those with osteoporosis [63]. However, no currently available anticancer treatment can treat osteoporosis. MLN4924 has shown potent in vitro antitumor activity against various cancers of the blood, liver, breast, gastrointestinal, colorectal, urinary tract, bone, and skin [28,64,65,66,67,68,69,70,71,72,73,74,75]. Several phase I clinical trials of MLN4924 for treating advanced solid tumors [26,76,77], metastatic melanoma [78], and hematological malignancies, including acute myeloid leukemia and high-grade myelodysplasia, lymphoma, and relapsing multiple myeloma, have reported favorable treatment responses and low toxicity [79,80,81,82]. Although, the concentration of MLN4924 injected into a mouse is hard to assess, regarding whether it did indeed inhibit neddylation in vivo. However, the concentration was referred to the previous study [83], and the significant improvement of in vivo results in the OVX and sRANKL and MLN group compared to the OVX and sRANKL group was indicating the effectivity of MLN4924. Our data also revealed that MLN4924 significantly inhibited RANKL-mediated osteoclastogenesis in vitro and prevented bone loss in the OVX and sRANKL and MLN group in vivo. Thus, MLN4924 might be useful for antiosteoporotic treatment in patients with cancer or those with a risk of osteoporosis or bone metastasis. However, rigorous basic research and clinical trials are required to corroborate these findings.

Currently, the most commonly used antiosteoporotic agents include bisphosphonate and denosumab, which inhibit osteoclasts to reduce bone resorption and increase bone mineral density. However, they are associated with excessive and prolonged suppression of bone turnover, resulting in an accumulation of microdamage and a reduction in intrinsic mechanical properties, delayed healing, atypical fracture, and osteonecrosis of the jaw [84,85,86,87,88,89,90,91]. To avoid suppression of bone turnover, modified forms of bisphosphonates, such as nitrogen-containing bisphosphonates, have been used to reduce the effect on osteoclast apoptosis. Studies have also proposed targeting the key enzymes of osteoclasts, such as cathepsin K or RANK-associated proinflammatory cytokines such as IL-20 antibody, which might share similar characteristics with MLN4924 [92,93,94]. By targeting the downstream of the RANKL pathway, MLN4924 seems to possess an advantage over the abovementioned antiosteoporotic agents, by not killing the osteoclasts but only reducing the differentiation. Therefore, MLN4924 may have potential antiosteoporotic effects with fewer adverse events.

This study has some limitations, which include an OVX/sRANKL-induced osteoporosis model that might undergo a rapid bone loss compared with the traditional OVX model. However, we focused on examining the pattern of the neddylation pathway in sRANKL-induced osteoclastogenesis, which may be better tested with this model to prevent other inflammatory factors such as TNF-α or LPS. Further research is required to identify the relationship between the TRAF6 and the neddylation pathway as well as to comprehensively assess the effect of the neddylation pathway on osteoblasts and osteocytes. Moreover, we found an interesting result that the measurement of trabecular thickness was not statistically different between the sham and OVX groups (Figure 6e) and was different from the other parameters. It was also observed in other studies [95,96,97,98,99,100], suggesting that trabecular thickness might be less sensitive than other parameters to bone turnover. We examined the impact of MLN4924 on an osteoblastic (MC3T3-E1) cell line, which showed no obvious effect on the calcification and its viability. Furthermore, no change in serum osteocalcin concentration was found in vivo. These results imply that MLN4924 mainly inhibits osteoclastogenesis at a low dose. We also did not compare the effect of MLN4924 with the currently available antiosteoporotic medication, thus precluding the determination of its efficacy. Future studies should evaluate this for determining its potency.

**Figure 10:**
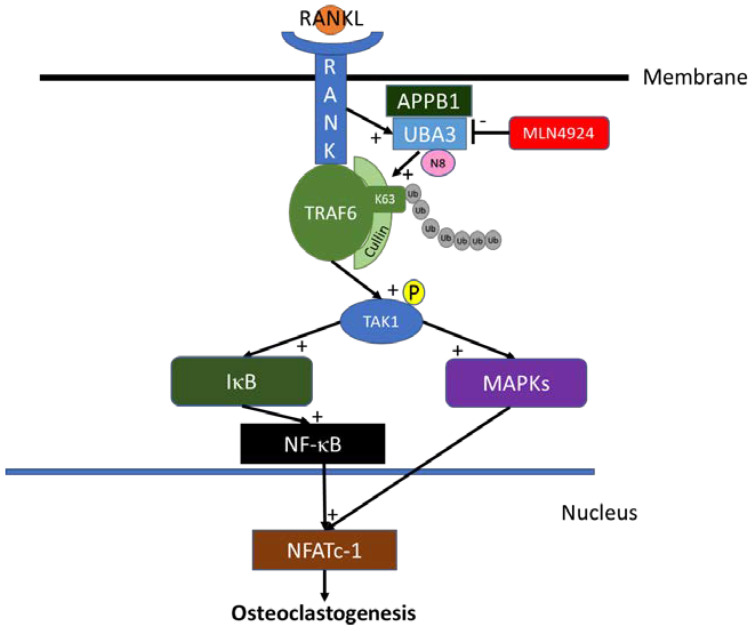
Possible mechanisms of MLN4924 treatment in sRANKL-induced osteoclastogenesis.

Taken together, the neddylation pathway is critical for sRANKL-induced osteoclastogenesis (Figure 10) and can be inhibited by an NAE inhibitor such as MLN4924.

## Data Availability

Not applicable.
